# Lack of access to health care for African indigents: a social exclusion perspective

**DOI:** 10.1186/1475-9276-12-91

**Published:** 2013-11-15

**Authors:** Werner Soors, Fahdi Dkhimi, Bart Criel

**Affiliations:** 1 Department of Public Health, Institute of Tropical Medicine, Antwerp, Belgium

**Keywords:** Access to health care, Indigence, Africa, Social exclusion

## Abstract

**Background:**

Lack of access to health care is a persistent condition for most African indigents, to which the common technical approach of targeting initiatives is an insufficient antidote. To overcome the standstill, an integrated technical and political approach is needed. Such policy shift is dependent on political support, and on alignment of international and national actors. We explore if the analytical framework of social exclusion can contribute to the latter.

**Methods:**

We produce a critical and evaluative account of the literature on three themes: social exclusion, development policy, and indigence in Africa–and their interface. First, we trace the concept of social exclusion as it evolved over time and space in policy circles. We then discuss the relevance of a social exclusion perspective in developing countries. Finally, we apply this perspective to Africa, its indigents, and their lack of access to health care.

**Results:**

The concept of social exclusion as an underlying process of structural inequalities has needed two decades to find acceptance in international policy circles. Initial scepticism about the relevance of the concept in developing countries is now giving way to recognition of its universality. For a variety of reasons however, the uptake of a social exclusion perspective in Africa has been limited. Nevertheless, social exclusion as a driver of poverty and inequity in Africa is evident, and manifestly so in the case of the African indigents.

**Conclusion:**

The concept of social exclusion provides a useful framework for improved understanding of origins and persistence of the access problem that African indigents face, and for generating political space for an integrated approach.

## Background

Access to health care for the African indigent populations has been an unsolved issue for several decades now. In the last quarter of the 20^th^ century, economic crisis, structural adjustment and the introduction of cost recovery for health services have exacerbated the problem but also triggered some compensatory policy responses [1,2], mainly in the form of fee exemption mechanisms. Ten years after the launch of the Bamako initiative^a^ in 1987, Gilson pointed out that those exemptions were rarely implemented, and–if implemented–usually ineffective in protecting the poorest [3,4]. Already in 1988–in a World Bank study on cost recovery in Senegal, Mali, Ivory Coast and Ghana–Vogel noticed: “It seems indeed ironic that almost every government official that was interviewed expressed concern for the poor in discussing changes in user charges, but at the same time freely admitted that the existing pattern of expenditure was extremely inequitable” [5].

At least in international circles, equity became a concern and by the end of the century, Stierle and colleagues provided a comprehensive review of concepts and policy measures specifically related to indigents in sub-Saharan Africa [6].^b^ First of all, the authors observed a widespread lack of precision on how to define indigence. They noted that the complex concept of indigence “covers, at least and interdependently, the dimensions of poverty and social exclusion” and that “the distinction between poverty and indigence seems particularly important for countries where the majority of people are poor”. Framing indigence as more than extreme poverty, Stierle and colleagues kept clear of the widespread confusion between the two notions. The authors also distinguished two fundamentally different approaches–which they termed ‘technical’ and ‘political’–for improving access to health care for the African indigents. On the one hand, the (more common) technical approach–within a depoliticised conceptualisation of indigence/poverty–reduces the challenge of indigents’ access to technical tasks, specifically to the identification of the indigents and effectively targeting services to them. On the other hand, the (less common) political approach seeks to reduce inequalities and to address the causes of poverty and exclusion, in order to improve the health status of the indigents [6]. Eventually–spelling out indigence as “the advanced state of poverty and social exclusion” and taking stock of the limited success of a segregated technical approach that had been unable to break the circle of poverty and to counteract social exclusion–Stierle and colleagues made a case for the integration of the technical and the political approaches [6].

Apposite as Stierle and colleagues’ proposal might have been, it has rarely been applied in practice. Until today, the issue of indigence has been mainly dealt with in a narrow social protection framework, increasingly based on targeted social assistance. Considerable efforts have been made to make targeting more effective through better identification of the poor, mainly at project [7,8], but also at national level [9,10]. Yet, for most African indigents, the results are as disappointing today as they were more than a decade ago. In West Africa–as Ridde pointed out–“30 years after Alma-Ata and 20 years after the B(amako) I(nitiative), the worst-off still do not have access to care” [11]. Such lack of progress should prompt all actors involved to seriously question mainstream policy.

Today, we have much more evidence than Stierle and colleagues could have had more than a decade ago to reassert that targeting is necessary and indispensable, yet insufficient on its own. The case for integrating the technical into a political approach is stronger than it ever was, but to bring about this policy shift remains an enormous challenge [12,13]. As Niño-Zarazúa and colleagues posited when contemplating the recent evolution of social protection in sub-Saharan Africa, “Getting the politics right may be as important, or even more important than getting the initial technical design of programs right”. Cautioning against optimism, they see the alignment of donors and national actors in favour of long-term social policy as a prerequisite–a condition that is still rarely fulfilled today [14].

In this paper we go one step further and argue that the analytical framework of social exclusion can contribute to bring about this desired change.

## Methods

We produce a critical and evaluative account of the literature on three themes (social exclusion, development policy and indigence in Africa) and their interface. As the aim of the underlying literature review is to actualise understanding of these complex themes and their possible interactions, our review method chosen is essentially narrative. We (re)introduce and appraise the concept of social exclusion, how it has been interpreted and how it can be construed, how it has been both valued and neglected, and how relevant it was and remains in the African context. We assert that purposeful analysis of the pathways of social exclusion within African societies–including within programmes and projects aimed at benefiting the excluded–strengthens the case and can generate political space for a comprehensive and integrated approach to social policy. Eventually, among other positive impacts of the latter, improvement in the conditions of the indigents is expected.

## Results

### Ups and downs of social exclusion

In most early reviews of social exclusion [15-19], the initial use of the term social exclusion is attributed to the French secretary of state Lenoir’s publication on ‘*les exclus*’, those unreached by a failing welfare state [20].^c^ With hindsight, this framing might have done more harm than good. Unlike the sociologist Elias, who had unravelled exclusion in relational terms a decade earlier [21], the politician Lenoir pinpointed categories of excluded in a very particular context. Matching seamlessly with a notion of residual social policy that soon would confirm its prominence in poor countries within their World Bank-backed Poverty Reduction Strategies [22], the focus on status and individuals more than on causes and processes provided a strong rationale for a targeted approach. In the case of the African indigents, this just meant more of the same: “individualized solutions to a general social problem” [6]. In addition, the emphasized conceptual origin of social exclusion in well-off welfare states, made it all too easy for the political elites in poor countries with high poverty rates to dismiss the concept entirely.

When social exclusion started permeating global vocabulary in the aftermath of the 1995 World Summit for Social Development [23], European policymakers had already adopted the concept. France had introduced its ‘*Revenu minimum d’insertion*’ in 1988, the United Kingdom set up a ‘Social Exclusion Unit’ in 1997, and Scotland a ‘Social Inclusion Division’ and ‘Social Inclusion Network’ in 1998, to name a few. These national initiatives differed markedly, not only because of different local contexts but also because of distinct understandings of what social exclusion is and how it should be addressed. Even so, they had one thing in common: a compensatory approach to deal with the consequences of social exclusion. The European ‘Poverty 3’ programme (1989-1994) consolidated the shift from poverty to exclusion in the social policy discourse. More recently, European policymakers started paying due attention to the structural, relational and personal dimensions of social exclusion, and to the experiences of the excluded themselves. In the more academic field, Nobel prize laureate Amartya Sen reframed Adam Smith’s not “being able to appear in public without shame” as “a capability deprivation that takes the form of social exclusion” [18].

When the International Institute for Labour Studies conducted a worldwide research project on social exclusion to inform the 1995 World Summit for Social Development (WSSD), social exclusion was still principally presented as “a state of affairs” [17] and “material poverty (…) as a particular form of social exclusion” [24]. The WSSD did a great job in putting both poverty and social exclusion on the global political agenda. Yet, the focus on exclusion as outcome more than process paved the way for a perpetuation of a residual poverty approach.

It took the research community some time to take a conceptual stand in favour of social exclusion as a process. Arjan de Haan was one of the first to stress that the relevance of the concept basically consisted in its focus on processes, causes and mechanisms that led to deprivation [15]. Amartya Sen took a similar stand in the debate: “The helpfulness of the social exclusion approach does not lie (…) in its conceptual newness, but in its practical influence in forcefully emphasizing–and focusing attention on–the role of relational features in deprivation” [18]. Eventually, researchers and policymakers in developed countries welcomed the shift in attention from outcome to process. International development actors were more reluctant to do so. Illustrative is the case of the UK Department for International Development (DFID) that in 2005 still described social exclusion as outcome as much as process [25].

While the World Bank’s Poverty Reduction Strategies and the United Nations’ Millennium Development Goals (MDGs) were not conducive to take a process approach on board, it was the WHO’s Commission on Social Determinants of Health that provided an opportunity to turn the tide. After a critical appraisal of a diversity of policies addressing social exclusion,^d^ the Commission’s Social Exclusion Knowledge Network (SEKN) presented a framework for understanding and tackling social exclusion as related to health inequalities that makes a conclusive case for a relational perspective. Within this perspective, the SEKN defined social exclusion as “dynamic, multidimensional processes driven by unequal power relationships interacting across four main dimensions–economic, political, social and cultural–and at different levels including individual, household, group, community, country and global levels. It results in a continuum of inclusion/exclusion characterised by unequal access to resources, capabilities and rights which leads to health inequalities” [26]. The Commission on Social Determinants of Health translated this definition in a principle of action: “Tackle the inequitable distribution of power, money, and resources–the structural drivers of (…) conditions of daily life–globally, nationally, and locally” [27]. The Commission thus opened a policy window for dealing with social exclusion processes to tackle health inequities.

Whether indeed appropriate action is been taken remains an open question. Where the need is greatest and specifically in Africa, as Marmot and colleagues observe [28], “countries such as Kenya and Mozambique have expressed real interest in social determinants of health, but we are unaware of what specific action might have followed”. All the same, a range of actors seems to be building up a momentum. In Kenya [29], Mozambique [30], Uganda [31], Zambia [32] and Zimbabwe [33], ‘Equity Watch’–a dynamic interface between national politics, researchers and activists to monitor progress and expose gaps in advancing health equity–emerged following the 2009 EQUINET (the Regional Network on Equity in health in East and South Africa) conference [34]. Zimbabwe pioneered this initiative with a first ‘Equity Watch’ already in 2008 [35] and kept on leading the process with a multi-sectorial priority-setting exercise for equity in health coverage and action on social determinants in February 2012 [36].

Besides, an emerging framework from a powerful international actor leaves room for hope: the World Bank’s Social Development Strategy [37]. Taking stock of progress since the 1995 World Summit for Social Development (WSSD) and reviewing its own involvement in social policy setting, the World Bank noted that “the WSSD commitment to support social integration was inexplicably left out of the MDGs”, that “the human development goals of the MDGs are necessary but not sufficient to achieve inclusive and sustainable development”, and that “there is a growing, albeit it somewhat grudging, acceptance of understanding and transforming power relations as an essential ingredient of development and poverty reduction” [38].

This led the Bank to pay more attention to structural inequalities, “perpetuated and reinforced by unequal relations in roles, functions, decision rights, and opportunities, which are bound in a web of interdependence”, and conceptualized as a vicious circle of structural inequality [39]. Such framing bears a welcome resemblance to the relational concept of social exclusion, as emphasized by the Commission on Social Determinants of Health.

### Social exclusion, developing countries, and development

The outcome vs. process debate was not the only difficulty that the concept of social exclusion was confronted with. Ever since social exclusion entered development discussions, the relevance of the concept as such and its applicability to developing countries met with scepticism. This was not unforeseen. In the document that introduced the concept to the 1995 World Summit, a prefatory caveat was included: “its relevance and value in a broader global context, and particularly in developing countries, has not yet been established” [17].

More than a few scholars questioned the relevance of the concept of social exclusion by pointing out its similarities with already existing concepts, such as multidimensional poverty or deprivation. Most academics however gradually accepted Sen’s argument for the intrinsic value of a social exclusion lens, independent from any claimed or disputed innovativeness. Exemplifying is the still critical yet balanced stand Saith took in 2001: the concept of social exclusion offers nothing new to research into causes, correlates and processes of poverty already conducted in developing countries, but might well help bringing these largely neglected studies to the fore [40]. In his comprehensive review, Estivill concluded: “The number of studies have to be increased (…) thereby creating a cumulative observatory (…) to influence and feed more general policies” [41].

While the amount and depth of empirical evidence for social exclusion in any society and throughout societies indeed steadily increased, translation into policy did not follow automatically. Lack of knowledge translation does not seem to be the issue. Nor can social exclusion be remedied by technical measures only. The fundamental issue is indeed that of the inequitable distribution of power, and thus of social exclusion itself as understood by the Commission on Social Determinants of Health [27]. Or as Labonté asked in no uncertain terms “how can one ‘include’ people and groups into structured systems that have systematically ‘excluded’ them in the first place?” [42]. We will elaborate on that paradox in our next section, dealing specifically with Africa. Here, we return to the applicability of the concept of social exclusion in the developing world as a whole.

Today, it can be said with good reason that social exclusion as such is truly universal, whereas its manifestations are obviously contextual. Or, as Bhalla and Lapeyre put it already more than a decade ago: “A plea for making exclusion a global concept is based on the assumption that the analytical concepts (…) are universal even if their operationalization in specific social and cultural contexts may be different” [43]. Following up on a worldwide research initiative, Gore and Figueiredo [24] both confirmed the universal presence of exclusion and its revealing range of contextual manifestations. In developing countries, exclusion from civil and political rights is often on a par with exclusion from social rights. Where citizenship remains shallow, states rarely compensate the unequal bargaining power of individual and group actors. Where societies are still predominantly agrarian, access to land can be more important than employment. And where poverty is the rule, social exclusion can be life-threatening.

Yet, where poverty is a mass rather than a minority phenomenon, the concept of social exclusion has been less applied. Specifically in sub-Saharan Africa, development agencies often preferred not to use the term because their local staff saw it less relevant for conditions of generalised poverty [44]. Such practice should be seen as short-sighted, if not detrimental. Above all, the neglect of a social exclusion perspective should be considered a missed opportunity in the sense that understanding social exclusion, as a process leading to poverty and inequity, is not less needed because one of the outcomes is more manifest than the other.

In sub-Saharan Africa, adherence to the poverty discourse prevented paying due attention to the fact that the region is not only poor, but also one of the most unequal in the world, surpassed only by Latin America [45]. Inequality in sub-Saharan Africa might actually be closer to that of Latin America than the figures may appear: the use of income instead of consumption measures in Latin America might overestimate inequality in the latter [46].

A social exclusion lens is in fact more needed in sub-Saharan Africa than elsewhere, precisely because the processes leading to poverty and inequity are more hidden than elsewhere. Besides, a social exclusion perspective can be helpful in drawing attention to the operation of social policy as a mechanism of exclusion itself [47].

Excessive reliance on targeting in poverty reduction and social security is a case in point. While targeting is acceptable (and needed) as add-on to universal policies, it is by and large ineffective on its own and full of drawbacks [22,26,48]. One particular inconvenience, as documented by Ellis [48] in pilot cash-transfer schemes for the destitute based on proxy indicators (Ethiopia) or on a 10%-of-the-population cutoff (Zambia and Malawi), is that of recipients ‘leapfrogging’ above the levels of consumption of non-recipients, thereby creating more social divisiveness and eroding support for the targeting schemes. One of the biggest disadvantages of sole targeting of the poor is social exclusion, as Simmel elucidated more than a century ago in Europe: by labeling the poor as needy and offering them assistance only, the assisted become socially even poorer, excluded from society [49]. In Africa, a classic and extreme example of such collateral exclusion-by-targeting is that of leprosy [50]: “It is neither leprosy nor poverty that kills the leper but loneliness” [47,51].

It should be clear by now that a social exclusion lens is as relevant to identify causes of poverty and inequity in developing countries as in developed countries. It should also be recognized how useful a social exclusion perspective is to counteract these causes, and to bring about genuine development.

Since the 1990s authors have supplied evidence for a chain of causality from human to economic development: social development contains crucial instruments for sustainable economic development [52,53]. At least as far as income inequality is concerned, we now know that high inequality is detrimental to economic development. This finding was first highlighted in a UNU/WIDER study [54], confirmed by the World Bank when laying out its Social Development Strategy [37], and corroborated in a 2010 UNRISD flagship report [55].

So why does social exclusion get so little attention where it is most needed?

### Social exclusion, Africa, and indigence

In Africa, the limited uptake of a social exclusion perspective–by international actors and local elites–is a multi-layered phenomenon. We already discussed the influential yet mistaken argument of social exclusion becoming less important in a context of widespread poverty. The disregard of social exclusion in Africa by non-Africans also goes hand in hand with a romanticised overestimation of African solidarity that overlooks material reciprocity as a main driver of solidarity in modern African society [56-60].

There is however no doubt that the common African is quite familiar with the existence and extent of social exclusion. In West Africa, the Malinke called both the poor and the indigents *fangantan*, those lacking power [61]. Wolof speakers in Senegal and the Gambia may still refer to indigents as *baadoolo*, those belonging to a social class with no power or strength (*doole*). They also use *baadoolo* for labelling individuals as egoistic, thus ungrateful to society. Kanuri speakers in Nigeria term the poorest of the poor *ngudi,* the unfortunate–judged outside the normal network of social relations and deemed not to be trusted [62]. In fact, in most African societies wellbeing is conceived in terms of social relations and kin networks, and a person’s place in them [63]. When exclusion is part of language, it is part of everyday life.

Political and development scientists show that social exclusion in Africa has increased, not decreased, over time. Inack and colleagues [64] describe how colonial rule reinforced exclusion based on ethnic identities in Cameroon. De Boeck [65] gives a detailed account of the same in Congo. Mamdani [66] sums up how throughout Africa the colonial state identified non-natives as races and natives as ethnicities. Races–white at the top–were considered a civilizing influence, and non-natives were governed through rights-based civil law. Ethnicities were considered in need of being civilized, and natives were governed through customary laws that enabled discriminatory power and left no room for civil rights. On top of existing intra-ethnic and reinforced inter-ethnic exclusion, colonial rule de facto excluded natives from society. In the early independence years, the nationalist aspiration of constructing a de-colonised identity was essentially the natives’ appeal for inclusion into the world of rights. To some extent and for some time, nationalism also masked social exclusion based on ethnic identities [13,66].

The subsequent nonfulfillment of the nationalist agenda, with state collapse at one extreme of the scale, had at least two interrelated consequences for the manifestations of social exclusion in post-colonial Africa: the absence of a ‘nationality of social exclusion’–defined by Gore [67] as “the significance of the nation State in the institutionalization of exclusionary practices”, and the perpetuation of exclusion through adverse incorporation–understood as the conditional inclusion of ‘outsiders’ in a closed relationship, by the privileged group in order to safeguard its monopolistic advantages [68-70]. Unaccomplished nation building shifted the locus of social exclusion away from the state. Lund [71] notes the rise of what he calls ‘twilight institutions’: “they are not the state but they exercise public authority”. Hagman and Péclard [72] observe a constant redefinition of statehood in post-colonial Africa, and a multitude of social actors who compete over the institutionalisation of power relations. Within such environment inclusion and exclusion are intertwined, inclusion more often than not entails adverse incorporation, and exclusion reproduces itself.

Adverse incorporation is as old as mankind: people who are excluded from one set of norms achieve some kind of inclusion once they abide by the unfavourable conditions of sustained exploitation [73]. At global level, its quintessence is the ‘untouchable’ Dalit in the South Asian caste system. In Africa, adverse incorporation is less explicit, much less recognized, but not to be overlooked.

Gore [67] points out how Africa’s unfulfilled ‘nationalisation’ of rights and obligations gives room to “multiple sites of exclusion and inclusion based on membership of a variety of shifting groups”, including clientelist networks. Wolfe [73] highlights how globalization and its concurrent of free-market based development give rise to clientelism and clannishness. Contemporary African society thus hosts multiple drivers of social exclusion and adverse incorporation. Still, their interpretation as problematic is uncommon, its presence often concealed or–in the view of the non-excluded–seen as a fact of life.

A case in point can be told from our own professional experience. In 2006 in Mali, when pre-testing an interview guide for a research on the influence of community health insurance (CHI) on the quality of care in primary health centres, we faced several obstacles. First, it was difficult to find among the villagers potential respondents who were unrelated to the village authorities, the health staff and the CHI leaders. Second, most of the villagers identified as unrelated to the same were reluctant to be interviewed. We felt thus pleasantly surprised when meeting, during a lunch break on the corner of the marketplace, half a dozen of women all unrelated to any village or health authority and very willing to speak out. We were offered a drink, had a chat with the women sitting on their mats, and promised them to come back with a voice recorder. We returned to the health centre and informed the health authorities gathered there about our pleasant encounter. Their reaction proved to be a cold shower. They told us that these women were repairers of gourds–a lower status occupation, were of improper behaviour and should be avoided at all cost. To get their point across, they ensured us that no man who would be seen sitting on a repairwoman’s mat would ever be allowed by the community to take a second wife, a mark of social distinction in the region. Besides, no policy actor would be interested in the repairwomen’s opinion, if they had any.

For a social scientist, this story would be a prime example of adverse incorporation: the women described are excluded from many domains, but allowed to live in the village as long as they accept their exclusions. For a global traveller, the story would bear a striking resemblance to the outcasting of Dalits from Hindu society. While several scholars [74-76] share a caste-based interpretation of West African societies in their academic writings, publicly referring to caste is unpopular in West Africa.

Lack of interest in the poor and aversion to excluded groups are by no means confined to village level. As Ridde [77] observes in a wider district setting, exclusion of the poorest from access to health care is customary and the problem of these worst-off is only dealt with after all other issues. At national level, Devereux and colleagues [78] note a disappointingly low uptake of donor-driven social protection pilot projects by African governments. Earlier, Hossain and Moore [79] documented how national political elites in developing countries are often unsupportive of, or in some cases hostile to, donor efforts to promote pro-poor policies.

These similar attitudes at all levels of society have one driver in common: a pervasive perception of a poor person as an unproductive good-for-nothing [14,80,81]. Partly overlapping with the political construction of the African ‘lazy man’ [81,82], it gives the non-poor a justification to exclude the poor. Among the poorest, the indigents are worst off. More often than not political elites consider them not only as unable to help themselves, but also as unable to benefit from poverty programmes [12,81].

While being antithetical to the pro-poor discourse of the donor community, the image of the unproductive poor finds confirmation in the still dominant economic model that promotes increased productivity within a competitive market [83] without worrying too much about those who simply cannot deliver to this paradigm. At the most, the indigents become passive recipients of handouts.

From here to a functioning welfare state based on social citizenship and de-commodified (not market-based) rights is obviously a long way. Yet it is precisely this arduous road that is needed to tackle social exclusion and that would offer the African indigents more than what Esping-Andersen [84] depicted as “a security blanket of last resort”.

Such road necessarily includes what Michielsen and colleagues term ‘the dimension of transformation’ of social protection in health: “transforming the social and institutional context (…) to counteract exclusion and deprivation of the right to health and quality care” [85]. To be substantive, transformation needs realisation of citizenship and political action at state level. As Mamdani has put it: “In the absence of a wider strategy of political change and social transformation, the empowerment of local communities can be of only limited and temporary significance” [86]. Social citizenship and transformation are the necessarily components of counteracting social exclusion, and are ultimately ‘distributive choices’ [87].

Today, mapping African states that fully realised social citizenship and inclusive social protection still results in a gloomy picture. Yet, clusters and pockets of progress are noticeable. This is certainly the case in Southern Africa, and to a much lesser extent in the rest of the continent. In Southern Africa, where urbanisation and industrialisation go hand in hand, substantial progress has also been made towards comprehensive social protection for over a decade. In West Africa, Ghana is the forerunner, since its introduction of a National Health Insurance Scheme (NHIS, 2005) and of a complementary cash-transfer programme (Livelihood Empowerment against Poverty, LEAP, 2008) [14].^e^ Both Southern Africa’s grant-based social protection schemes and Ghana’s efforts are by and large tax-funded, delivered by public agencies and enshrined in legislation. And, as Niño-Zarazúa and colleagues [14] note, “the connection between program entitlements and citizenship rights is to the fore”.

The importance of social movements as a driving force for transformation and progress cannot be understated. Again, this is especially noticeable in the recent history of Southern and South Africa [14,88]. Taking South Africa as an example, it is clear that social movements have been instrumental in both the transition from the pre- to the post-apartheid state and in the shaping of social policies in the post-apartheid era. The post-apartheid social movements–in the words of Habib [89]–launched “a fundamental challenge to the hegemonic political and socio-economic discourse that defines the prevailing status quo”.

A lesser, still barely traceable impact of social movements is to be found in contemporary West African anti-slavery groups that–despite slavery being a taboo subject–are struggling “to establish new social values according to which people of slave descent should equally be able to access resources and political offices” [90].

In addition to social movements as a political driving force against social exclusion and for progress towards comprehensive social protection, we identify the fairly recent emergence of South-South cooperation as potentially favourable for progress in Africa. Unlike North-South donor aid, which is all too often restricted to project activities that fail to make substantive transformation at national level, South-South cooperation is already positively impacting national social policies in Africa. The prime example is the contribution of the Brazil-Africa cooperation programme on social development and social protection, particularly to the design and follow-up of the Ghanaian Livelihood Empowerment against Poverty (LEAP) initiative. What distinguishes this South-South exchange of experience from North-South aid is indeed its remarkable uptake by national policymakers. The Ghanaian government embedded LEAP in a comprehensive social protection framework and is its main financing source.

## Conclusion

Both the political action of African social movements and a changing international development spectrum are potentially beneficial for a move towards more comprehensive social protection in Africa, and for tackling social exclusion. This should eventually and sustainably benefit the indigents, who are the top of the iceberg of a larger phenomenon. As philosopher Alain Badiou (quoted in [88]) put it: “Today the great majority of people do not have a name; the only name available is ‘excluded’, which is the name of those who do not have a name”.

The tide is high, and the time is right to bring back to mind Stierle and colleagues’ [6,91] call for the integration of technical and political approaches in support of the African indigents. The concept of social exclusion provides a useful framework for this much needed action.

## Endnotes

^a^This joint WHO/UNICEF initiative sought to improve African primary health care by making essential drugs available through self-financing at district level [92]. While the ultimate goal remained unreached, the Bamako initiative did provide a justification for cost recovery for decades to come. For a critical early evaluation of the Bamako initiative, see [93].

^b^Stierle and colleagues’ article drew upon an extensive literature review published earlier by the German Technical Cooperation [91].

^c^Thereby overlooking–amongst others–Ambedkar’s substantial conceptualisation (1916 onwards) of social exclusion in the Indian caste system [94], as well as the Latin American debates (1960s onwards) on marginalisation as a driver of social exclusion [95].

^d^This exercise lasted from 2006 to 2008 and resulted in a series of 16 background papers covering four world regions [26].

^e^Interestingly, Ghana is the only African country that dedicated an entire Human Development Report to social exclusion [96].

## Competing interests

The authors declare that they have no competing interests.

## Authors’ contributions

WS conceived the literature review and analysis, and produced the first draft of the manuscript. FD and BC complemented the analysis and commented on the draft manuscript. All authors read and approved the final manuscript.
